# Prussian Blue Nanoparticle-Labeled Mesenchymal Stem Cells: Evaluation of Cell Viability, Proliferation, Migration, Differentiation, Cytoskeleton, and Protein Expression In Vitro

**DOI:** 10.1186/s11671-018-2730-z

**Published:** 2018-10-22

**Authors:** Jirui Wen, Zhiwei Zhao, Ruijie Tong, Liwei Huang, Yali Miao, Jiang Wu

**Affiliations:** 1 West China School of Basic Medical Sciences & Forensic Medicine, Sichuan University, No17, 3rd section, Renmin Nanlu Road, Chengdu, 610041 Sichuan China; 2 College of Pharmaceutical and Biological Engineering, Shenyang University of Chemical Technology, Shenyang, China; 3 West China School of Stomatology Medicine, Sichuan University, Chengdu, China; 4 Department of Gynecology, West China Second University Hospital, Sichuan University, Key Laboratory of Birth Defects and Related Diseases of Women and Children (Sichuan University), Chengdu, China; 5 Deep Undergroud Space Medical Center, West China Hospital, Sichuan University, No.17, 3rd Section, Renmin Nanlu Road, Chengdu, 610041 Sichuan China

**Keywords:** Prussian blue nanoparticles, Mesenchymal stem cells, Magnetic resonance imaging, Cytotoxicity

## Abstract

Mesenchymal stem cells (MSCs) have been used for the treatment of various human diseases. To better understand the mechanism of this action and the fate of these cells, magnetic resonance imaging (MRI) has been used for the tracking of transplanted stem cells. Prussian blue nanoparticles (PBNPs) have been demonstrated to have the ability of labeling cells to visualize them as an effective MRI contrast agent. In this study, we aimed to investigate the efficiency and biological effects of labeled MSCs using PBNPs. We first synthesized and characterized the PBNPs. Then, iCELLigence real-time cell analysis system revealed that PBNPs did not significantly alter cell viability, proliferation, and migration activity in PBNP-labeled MSCs. Oil Red O staining and Alizarin Red staining revealed that labeled MSCs also have a normal differentiation capacity. Phalloidin staining showed no negative effect of PBNPs on the cytoskeleton. Western blot analysis indicated that PBNPs also did not change the expression of β-catenin and vimentin of MSCs. In vitro MRI, the pellets of the MSCs incubated with PBNPs showed a clear MRI signal darkening effect. In conclusion, PBNPs can be effectively used for the labeling of MSCs and will not influence the biological characteristics of MSCs.

## Background

Mesenchymal stem cells, a type of adult stem cell, have the capacity of anti-inflammatory, regenerative potential and can migrate into injured tissues to aid the recovery of damaged function [1]. They can differentiate into multiple cell types under the specific microenvironment and are easily collected from adult and fetal tissue [2]. Thus, mesenchymal stem cells (MSCs) have been used for regenerative medicine and oncology therapy as a promising tool due to these excellent properties [3, 4]. However, the fate of MSCs after transplanting into the body remains unclear, and non-invasive MSC tracking is necessary in vivo for evaluating the efficiency of transplantation and their fate, properties, and localization [5]. Recently, magnetic resonance imaging (MRI) as an effective technology has been widely obtained to research the structural and functional information of mesenchymal stem cells in vitro and in vivo [6].

In the past years, multiple nanoparticles were used for labeling MSCs as a promising tool for non-invasive imaging of cells to record their distributions and fate in vivo and in vitro, and even used for the treatment of tumors [7]. For example, superparamagnetic iron oxide (SPIO) nanoparticles and quantum dots (QDs) have been used for labeling cells for many years [8, 9]. And fluorescent magnetic nanoparticles (FMNPs) were used for labeling MSCs to realize the targeted imaging and synergistic therapy of gastric cancer cells in vivo [10]. For these novel labels, a careful and complete analysis of cell toxicity is needed because everything has a toxic dose and may perturb downstream cell function [11]. For example, the MRI contrast iron oxide nanoparticles was attributed to the generation of ROS and may cause cell death [12].

Recently, Prussian blue nanoparticles (PBNPs) have been demonstrated that they have potential to be an MRI contrast agent [13–15]. Prussian blue, considered as a practical, economical, safe, and environmently friendly drug, has been approved by the US Food and Drug Administration (FDA) in clinic to therapy the radioactive exposure. Importantly, the PBNPs were highly dispersible and stable in both water and biological mimic environments such as blood serum without the appearance of aggregation within 1 week [16], and have good photothermal stability that could reuse the PBNPs during practical applications [17]. For example, Liang et al. [13] firstly demonstrated that PBNPs with strong absorption in the NIR region can be used as an excellent contrast agent to enhance photoacoustic imaging. PBNPs with uniform size and good colloidal stability can be fabricated from low-cost chemical agents by an easy way.

The PBNPs have been used for labeling some tumor cells as the MRI agent in research [18], but little studies were reported on the application of PBNPs in the MSCs. Here, we report that PBNP-labeled mesenchymal stem cells exhibited normal cell viability, proliferation, migration, cytoskeleton, differentiation, and protein expression in vitro. Further work is needed if this is a reality in vivo and to make sure if directed intralesional delivery of PBNP-labeled MSCs is as critical as cell tracking thought.

## Methods

### Cell Culture

Mouse MSCs C3H10T1/2 were obtained from Nanjing KeyGen Biotech. Inc. Cells were cultured in Dulbecco’s modified Eagle’s medium (DMEM; Hyclone, USA) supplemented with 10% fetal bovine serum (FBS; Israel) and 1% penicillin-streptomycin (Hyclone, USA) at 37 °C with 5% CO_2_ saturation, and the medium was changed every 3 days. After four passages, the cells were used for experiments.

### Preparation of PBNPs

In a typical synthesis, 2.5 mmol of citric acid (490 mg) was first added to a 20 mL 1.0 mM aqueous FeCl_3_ solution under stirring at 60 °C. To this solution was dropwise added a 20 mL 1.0 mM aqueous K_4_[Fe(CN)_6_] solution containing the 0.5 mmol of citric acid (98 mg) at 60 °C. A clear bright blue dispersion formed immediately. After 30 min, the solution was allowed to cool to room temperature with the stirring continued for another 5 min at room temperature. Then, an equal volume of ethyl alcohol was added to the dispersion and centrifuged at 10,000 rpm for 20 min to result the formation of a pellet of nanoparticles. The latter was separated again by the addition of equal volume of ethyl alcohol and centrifugation.

### Characterization of PBNPs

The infrared spectroscopy of the synthesized PBNPs were measured using an infrared spectrophotometer (IR; Thermo Fisher Nicolet IS10). Morphology of the synthesized PBNPs was examined by transmission electron microscopy (TEM; JEM 2100F). Field-dependent magnetization of PBNPs was researched using a vibrating sample magnetometer (VSM; Lakeshore 7307). X-ray diffraction (XRD) analysis was performed using Bruker D8 ADVANCE A25X (XRD). The polydisperisty index of the PBNPs was determined by Zetasizer Nano ZS.

### Intracellular Distributions of PBNPs and Ultrastructure of Labeled C3H10T1/2 Cells

Transmission electron microscopy (TEM) was performed to assess the intracellular distributions of PBNPs. After the medium was removed, cells were rinsed with PBS and then digested with 0.25% trypsin. Then, the cells were transferred to a 1.5-mL EP tube to be centrifuged (2000 rpm, 5 min). Then, the supernatant was removed and the cells were fixed by 0.25% glutaraldehyde and 1% osmium acid. After cells were rinsed again, the cells were dehydrated in 50% ethanol, 70% ethanol, 90% ethanol, 90% acetone, and 100% acetone for 20 min each. Then, cells were imbedded at 4 °C overnight. Then, 3% uranium acetate-citrate double staining in sections was performed. Finally, images were collected by TEM.

Scanning electron microscopy (SEM) was done to assess the ultrastructure of labeled C3H10T1/2 cells. After the medium was removed, cells were rinsed with PBS and fixed with 3% glutaraldehyde precooling 4 °C overnight. Then, the cells were rinsed twice with PBS and fixed with 1% osmic acid 4 °C for 1 h. After C3H10T1/2 cells were rinsed again, the cells were dehydrated in ascending graded alcohols (30% ethanol, 50% ethanol, 70% ethanol, 80% ethanol, 90% ethanol, 95% ethanol, and 100% ethanol) for 2 × 10 min each. Then, the cells were immersed in 70%, 80%, 90%, 95%, and 100% acetonitrile solution for 15 min each. Then, vacuum drying and spray coating of gold were performed. Finally, images were collected by SEM.

### Cell Viability Analyses of the PBNPs

Cell viability was evaluated using the MTT (Sigma, USA). Cells were seeded into 96-well plates at 1 × 10^3^ cells per well at 37 °C with 5% CO_2_ atmosphere. After incubation overnight, the culture medium was replaced by 100 μL fresh medium containing different concentrations of PBNPs (0, 5, 10, 20, 40, and 80 μg/mL), and then cells were cultured for another 1 to 3 days. The culture medium was removed, and the cells were incubated with 20 μL of MTT (5 mg/mL) at 37 °C for 4 h. The precipitated violet dye crystals were dissolved in 150 μL of dimethyl sulfoxide (DMSO; Sigma-Aldrich, USA) for 10 min by shaking gently. The optical density (OD) value was measured at a wavelength of 490 nm using a microplate reader. The results of cells were expressed as percent viable cells.

### Proliferation Assay

Comparison with MTT, MTS, WST-1, and XTT, RTCA allows the analysis of the whole period of the experiment and does not require the labeling that negatively affects cell culture experiments [19]. So the xCELLigence system (Roche/ACEA Biosciences) was used to measure cell proliferation in real time. Briefly, cells were seeded into E-Plate-16 (ACEA Biosciences, Inc. San Diego, USA) at 5 × 10^3^ cells per well with 150 μL complete medium. After growing 24 h, the medium was replaced by fresh medium containing various concentrations of PBNPs and incubated for another 96 h. This system measured the electrical impedance, which was created by cell attachment on the microelectrode-integrated cell culture plates [20], to provide the quantitative information about the cell number and viability in real time by the RTCA-DP instrument [21]. Cellular proliferation was measured periodically every 15 min for the following 4 days.

### Real-Time Monitoring of Cellular Migration

C3H10T1/2 cell migration was measured using a real-time cell invasion and migration (RT-CIM) assay system (ACEA Biosciences, Inc. San Diego, USA). Cells have the ability of increasing the impedance to hence “Cell Index” read-outs when they contact and adhere to the sensors due to the cell migration from the upper chamber into the bottom chamber through the membrane. Simply, cells were seeded in the upper chamber at a density of 4 × 10^4^ per well in serum-free medium in the presence of various concentrations of PBNPs. The lower chambers of CIM plates were filled with 165 μL complete medium containing 10% FBS. Cell migration was monitored by RTCA DP instrument every 10 min for a period of 100 h. Cell index (CI) was used to reflect the results. The value of CI was derived from the change in electrical impedance as the living cells interact with the biocompatible microelectrode surface in the microplate well to effectively measure cell number, shape, and adherence. The more the number of migrated cells, the larger the cell index.

### Cellular Migration Investigation via Transwell Assay

After being cultured with different concentrations of PBNPs for 48 h, the cells with density of 2 × 10^6^ cell/cm^2^ were cultured in a Transwell chamber (8 μm pore size; BD FalconTM, USA) for 24 h at 37 °C and 5% CO_2_. After culturing, the inner chamber was cleaned, and the migrated cells on the bottom of the chamber was fixed and stained with 0.1% crystal violet. Each step was followed by washing with PBS for 5 min three times. The migrated cells were photographed at different fields of view using an inverted phase-contrast microscope (CK2, Olympus, Japan).

### In Vitro Cell Differentiation

C3H10T1/2 cell-labeled or unlabeled PBNPs were induced to differentiate into two downstream cell lineages of adipocytes or osteocytes. After cells reached confluence in a six-well plate, cells were cultured in in osteogenic induction medium (10% FBS/DMEM containing 10 nM dexamethasone, 50 μM ascorbic acid, 10 mM β-glycerophosphate, Sigma) or adipogenic induction medium (10% FBS/DMEM containing 1 μM dexamethasone, 0.5 mM isobutylmethylxanthine, 10 μM insulin, Sigma). After 3 weeks, the cells were washed with PBS, fixed with 4% polyoxymethylene, and stained with Alizarin Red or Oil Red O (Sigma). The induced cells were photographed at different fields of view using an inverted phase-contrast microscope (CK2, Olympus, Japan).

### Immunofluorescence Assay for F-actin Visualization

MSCs were cultured with various concentrations of PBNPs at a 24-well plate for 48 h. The cells were fixed with 4% paraformaldehyde for 10 min, permeabilized with 0.2% Triton-100 for 5 min, blocked with 1% BSA in PBS for 30 min at room temperature, and then cultured with phalloidine (1:100, Thermo Fisher Scientific, USA) and DAPI (1:800, Thermo Fisher Scientific, USA) for 30 min at room temperature. Fluorescence microscopy was performed on a Nikon eclipse Ti-S microscope with NIS elements software

### Protein Expression by Western Blot Analysis

The protein expression of the cells was evaluated via Western blot analysis. The MSCs were cultured with the medium containing different concentrations of PBNPs (0, 25, 50 μg/mL) on six-well plates for 24 h, washed twice with ice-cold PBS, and scraped in 100 μl PIPA buffer (Beyotime) containing protease inhibitors and sodium orthovanadate (Beyotime, China). After 30 min, the samples were centrifuged at 14,000 rpm for 10 min at 4 °C, then the protein concentrations of the samples were determined using BCA kit (Beyotime, China). The same amount of proteins were electrophoresed in 10% SDS-PAGE gels (Beyotime, China) and transferred to PVDF membrane (GE Healthcare). The membranes were blocked with 5% milk in Tris-buffered saline with Tween20 (TBST) at room temperature for 2 h and then incubated with anti-β-catenin (1:1000, CST, USA), anti-vimentin (1:1000, Abiocode), and anti-β-actin (1:1000, CST, USA) overnight at 4 °C. The membranes were washed three times for 5 min each and then incubated with the appropriate secondary antibodies for 2 h at room temperature. Signals were detected with ECL and ECL-plus (Beyotime, China) and exposed to Molecular Image® ChemiDoc™ XRS+ system (Bio-Rad Inc., USA) with Image Lab™ Software using enhanced chemiluminescence.

### Cellular Imaging Investigation of Cellular Labeling Efficiency via MRI

MSCs were treated with different concentrations (25 and 50 μg/mL) of PBNPs, and the control cells were cultured with completed medium without PBNPs for 48 h, washed three times with PBS buffer, trypsinized, collected, and then embedded in 1 mL 1% (*w*/*v*) agarose solution for imaging studies. Additionally, the MSCs labeled with 50 μg/mL PBNPs were induced to osteogenic differentiation for 14 days, then were examined the MRI signal effect. The T2-weighted imaging was performed using an inversion recovery gradient echo sequence with TE = 23 ms, TR = 400 ms, NEX = 2.0, a slice thickness of 2 cm, a FOV of 20 × 20 cm, and matrix size of 384 × 256.

### Statistical Analysis

The results were expressed as mean ± SD of at least three independent experiments performed in triplicate. Treatment groups were compared using one-way analysis of variance (ANOVA) and Student’s *t* test was used. *p* < 0.05 was accepted as a significant difference.

## Results and Discussion

### PBNP Characterization

**Fig. 1:**
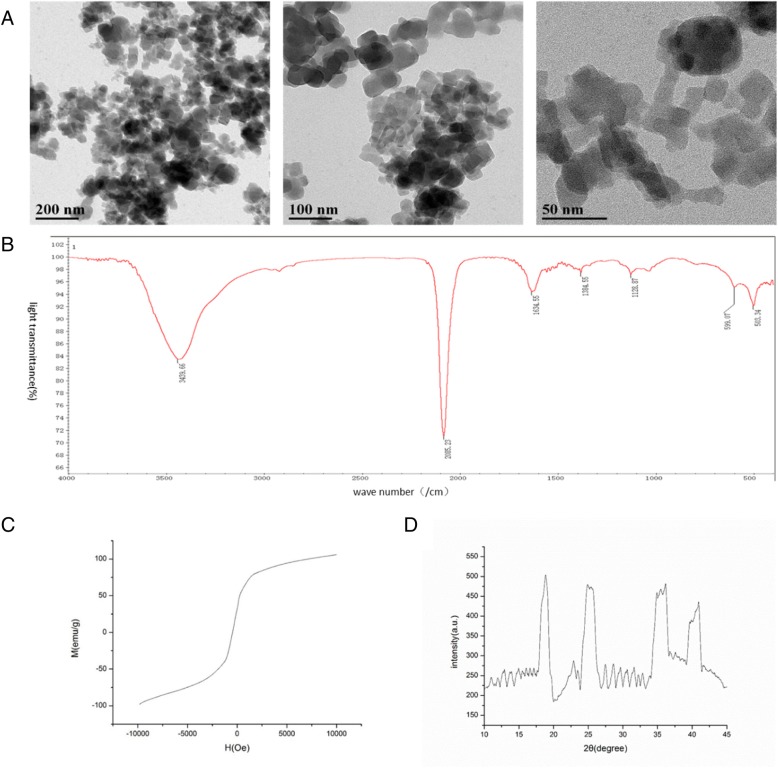
PBNP characterization. **a** Morphology of the PBNPs. **b** UV-vis absorbance spectra of the PBNPs. **c** Field-dependent magnetization of PBNPs. **d** XRD pattern of PBNPs

Transmission electronic microscopy (TEM) was performed to characterize the PBNPs (Fig. 1a), which have a diameter of 20–25 nm. For the morphology, the PBNPs showed a cuboidal structure. Figure 1b shows the infrared spectroscopy of the synthesized PBNPs. The PBNPs exhibited a typical absorption peak of Fe^3+^-CN around 2085.23 nm, which was in agreement with that of PBNPs. Field-dependent magnetization measurement was further used to study the magnetic properties of the PBNPs. Figure 1c shows magnetization curves of the PBNPs at room temperature, which demonstrated superparamagnetism of the PBNPs. Figure 1d shows the diffraction peaks at 200, 220, 400, and 420, which corroborated with the XRD pattern of PBNPs. Additionally, the polydisperisty index of PBNPs was 0.16, which indicated a uniform particle size distribution.

### Cellular Uptake and Cytotoxicity of PBNPs

**Fig. 2:**
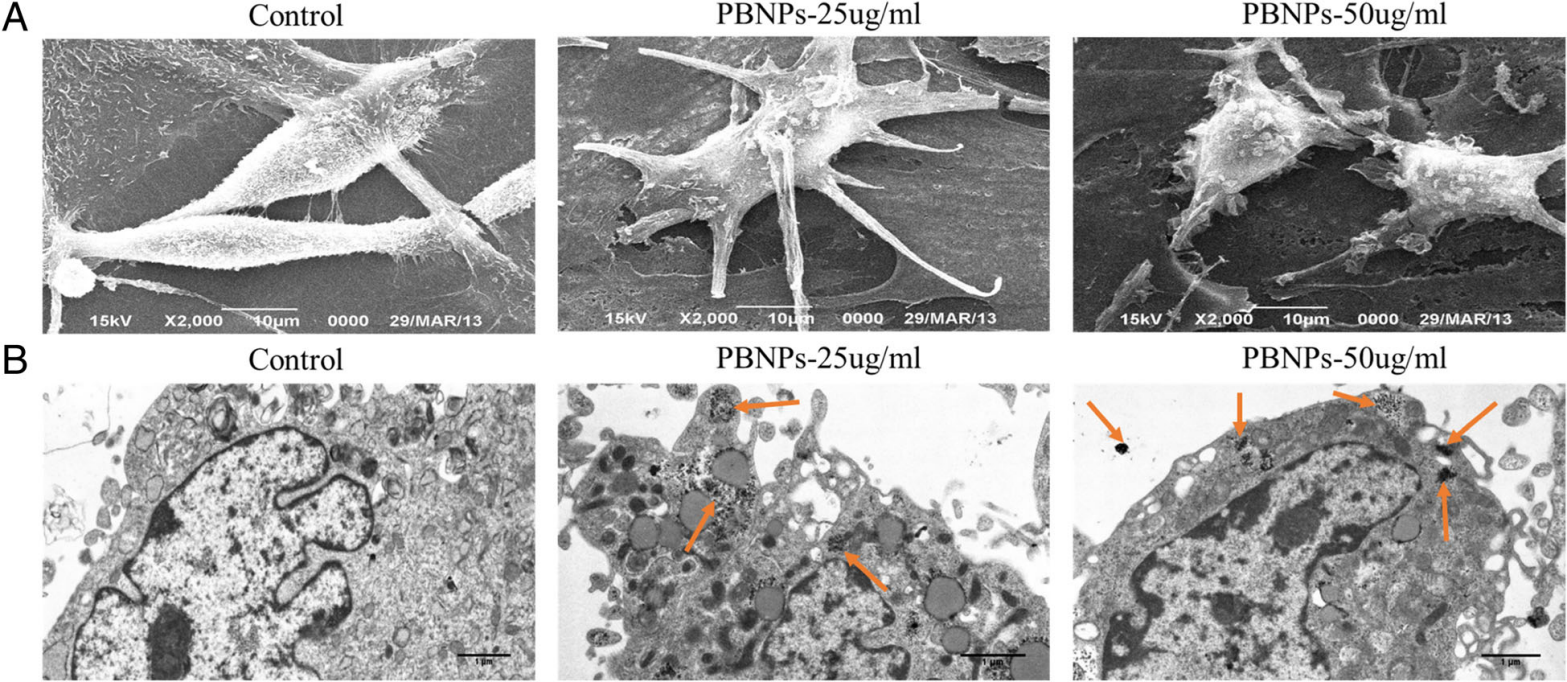
SEM and TEM images of the C3H10T1/2 cells after the incubation with different concentrations of PBNPs for 48 h. **a** SEM images. **b** TEM images. ↑, intracellularly distributed PBNPs

To further confirm the cellular uptake of the PBNPs to MSCs, cellular micromorphology of the above C3H10T1/2 cells treated with and without the PBNPs was studied. Figure 2 shows SEM and TEM images of C3H10T1/2 cells after the incubation for 48 h with and without the PBNPs. From the SEM images, the ultrastructure of the labeled C3H10T1/2 cells did not have obvious changes when compared with the control C3H10T1/2 cells. From the TEM images, the control C3H10T1/2 cells without the incubation with the PBNPs exhibited a typical cellular micromorphology with obvious cellular microstructures. Yet, after incubation with the PBNPs, random distribution of the PBNPs was clearly observed in the cytoplasm of the C3H10T1/2 cells. And some PBNPs appeared to be localized in vesicles within the cytoplasm of the cells. Although the random distribution of the PBNPs was observed in the cytoplasm of the C3H10T1/2 cells, the exact mechanism of intracellular uptake was unclear. We propose that the internalization of the PBNPs in C3H10T1/2 cells may occur via a similar mechanism as the previous study demonstrated, which had reported that different inorganic nanoparticles including Prussian blue–Poly(l-lysine), gold, silver, and metaloxides can be readily taken up by cells via endocytosis [15, 22, 23].

**Fig. 3:**
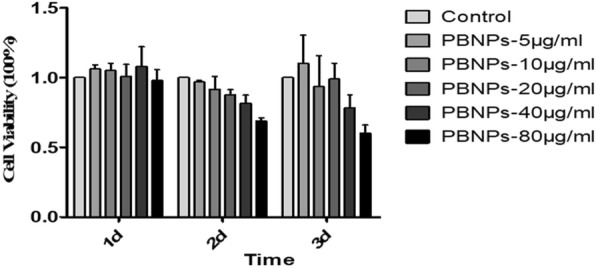
The viability of C3H10T1/2 cells in the presence of various amounts of PBNPs as determined by the MTT method

**Fig. 4:**
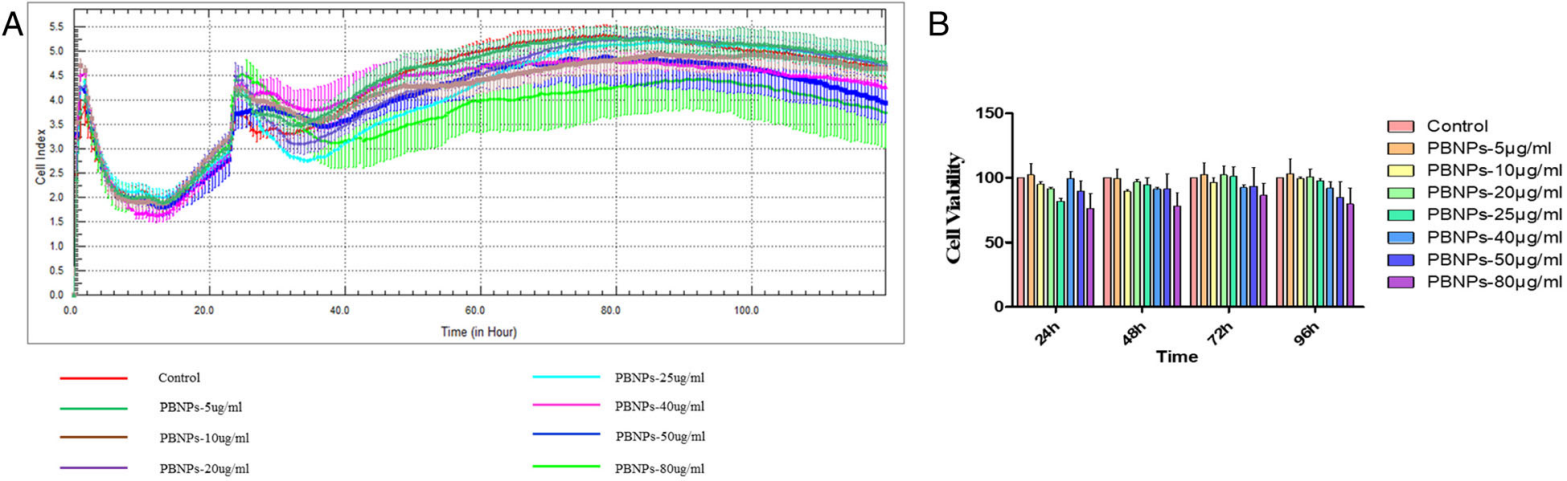
The proliferation of C3H10T1/2 cells in the presence of various amounts of PBNPs as determined by RT-CIM assay. **a** The growth curves of C3H10T1/2 cells. **b** The cell viabilities of C3H10T1/2 cells after treating in 24, 48, 72, and 96 h

To evaluate the cytotoxicity and the cell viability assay in MSCs, MTT method was performed. The cells were incubated for 1 to 3 days at 37 °C under 5% CO_2_ with various concentrations of PBNPs suspended in DMEM. Three independent trials were conducted, and the averages and standard deviations were reported. Figure 3 shows that the viability of MSCs treated with PBNPs (5, 10, 20, 40, 80 μg/mL) was relative to the control cells at 24 to 72 h, respectively. The results indicated that the PBNPs were non-toxic to cells treated with the same amount of PBNPs as MTT. Furthermore, a real-time proliferation assay using the xCELLigence instrument was used for investigating the growth curves of MSCs. Results showed that the growth curves of MSCs were not significantly influenced by these concentrations of PBNPs (Fig. 4a), and the cell viabilities were counted and showed in Fig. 4b after treating in 24, 48, 72, and 96 h. These results suggest that PBNPs have no effect on the proliferation of MSCs.

### Cell Migration Capability

**Fig. 5:**
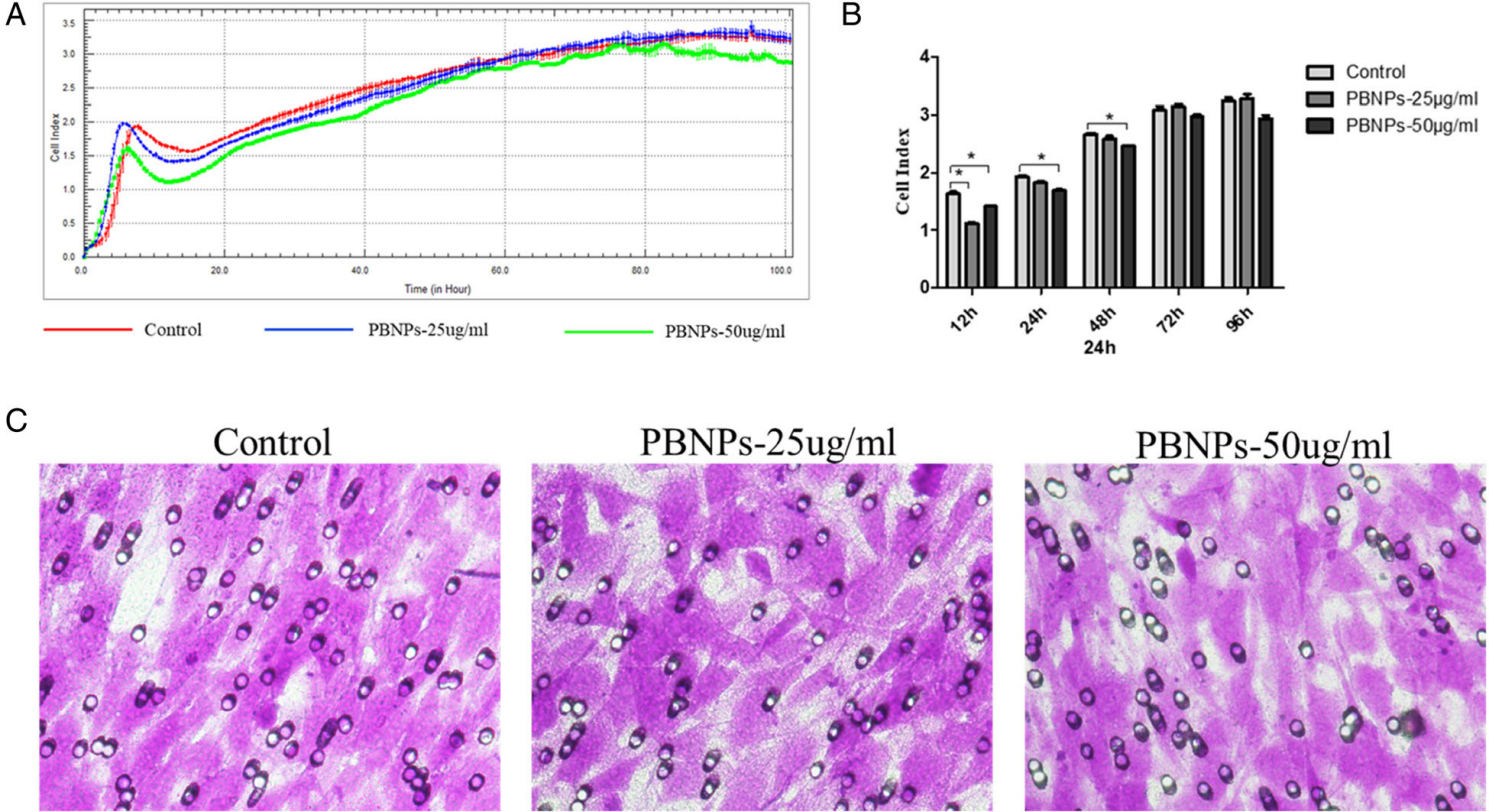
The migration of C3H10T1/2 cells in the presence of various amounts of PBNPs. **a** The migration of C3H10T1/2 cells determined by RT-CIM assay. **b** The cell index of C3H10T1/2 cells after treating in 12, 24, 48, 72, and 96 h. **c** The migration of C3H10T1/2 cells determined by Transwell assay (magnification × 200)

Migration of MSCs treated with various concentrations of PBNPs was tested using Transwell assay and a new technique, RT-CIM assay system. From the Transwell assay, the labeled cells showed no obvious changes in migration. By using the RT-CIM assay system, cell migration was monitored in real time, which reflected a more accurate data and could predict cell migration capability more accurately. From the RT-CIM assay system, although at the beginning of the labeling, the labeled cells migrated slowly than the unlabeled cells. But at 72 and 96 h, there was no significant difference in cell migration between labeled cells and unlabeled cells, indicating that high concentrations of PBNPs did not affect MSC motility (Fig. 5).

### In Vitro Cell Differentiation

**Fig. 6:**
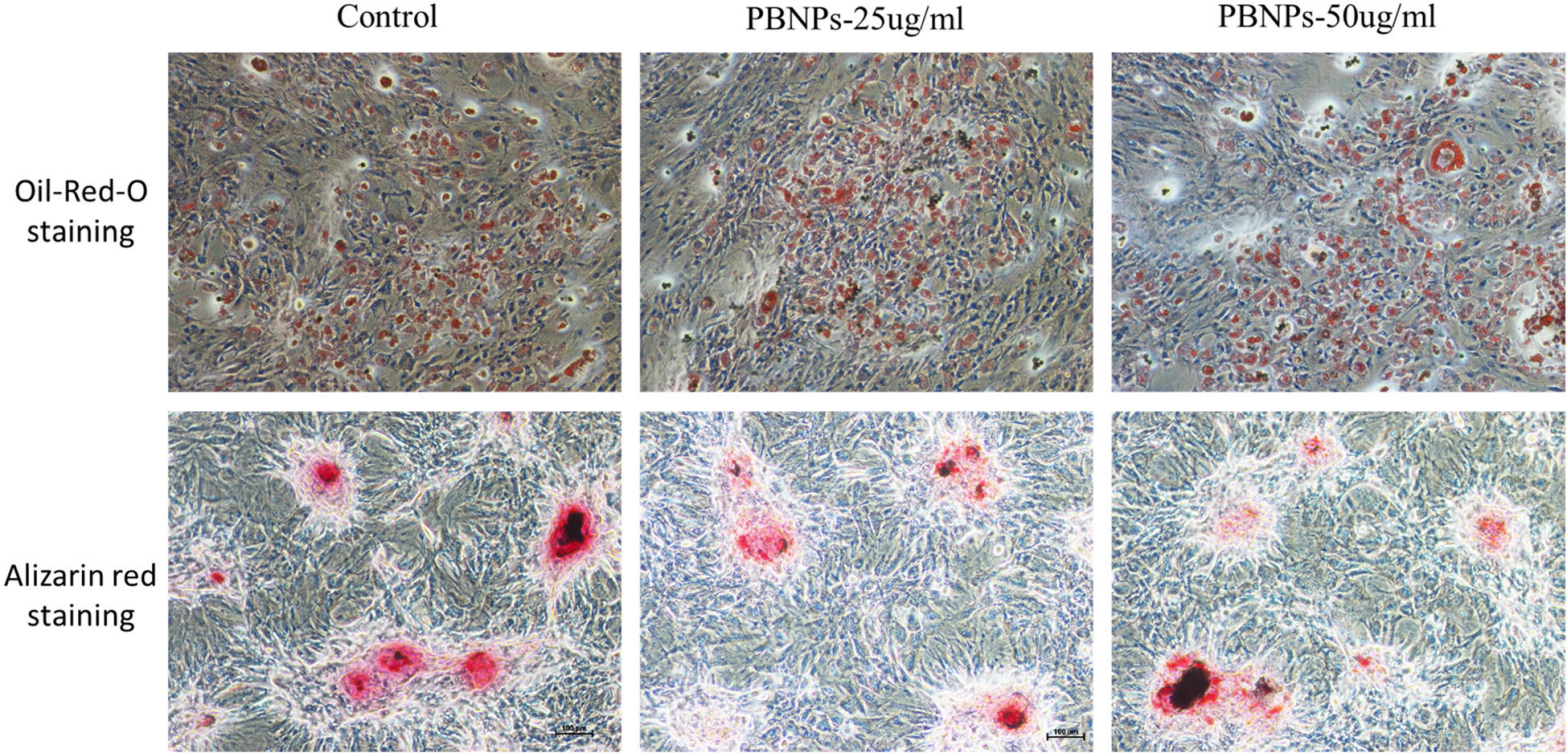
In vitro cell differentiation of cells with or without PBNPs. Oil Red O staining for adipocyte and Alizarin Red staining for osteocytes. Images were obtained 3 weeks after labeling (magnification × 200)

The pluripotency of labeled and unlabeled MSCs was investigated by Alizarin Red and Oil Red O staining. Figure 6 shows that labeled MSCs can be successfully differentiated into adipocytes and osteocytes as the unlabeled MSCs did. These results suggest that the PBNPs did not interfere with the cells’ differentiation capacity, which kept the pluripotency of labeled MSCs.

### Influence of the Labeling PBNPs on the Cytoskeleton

**Fig. 7:**
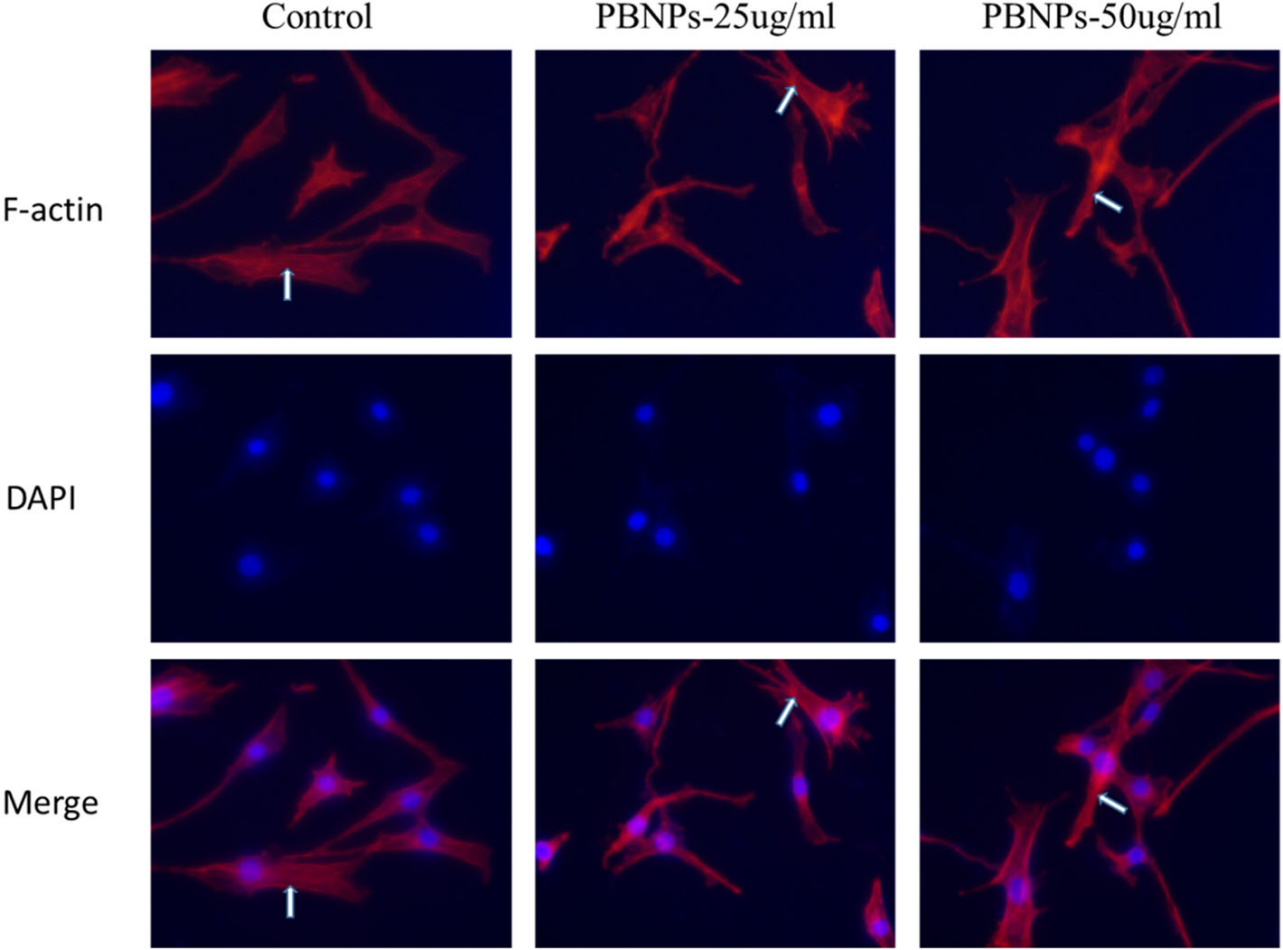
Immunofluorescence of C3H10T1/2 cells in the presence of various amounts of PBNPs for 48 h (magnification × 400). ↑, cytoskeleton

To investigate the effect of PBNPs on the cytoskeleton of MSCs, immunofluorescence assay of F-actin was used. The phalloidin staining shows no alteration of the red actin filaments of the cytoskeleton after labeling for 48 h compared with unlabeled MSCs. A comparison of the integrity and distribution of actin filaments in the labeled and unlabeled cells for 48 h revealed no alterations (Fig. 7).

### Western Blot Analysis

**Fig. 8:**
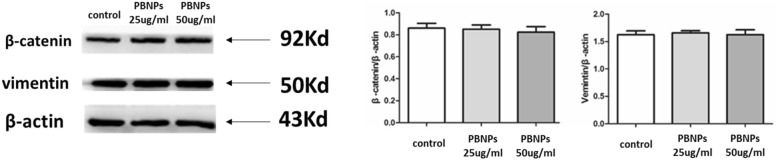
Western blot shows the expression of β-catenin and vimentin of MSCs treated with and without PBNPs for 48 h. The level of β-catenin and vimentin was quantified by software ImageJ

Wnt signaling pathways play an important role in the regulation of cell proliferation, differentiation, apoptosis, tissue formation, and the stem cell fate [24]. So, β-catenin is the functional protein of MSCs. Additionally, the vimentin is the mesenchymal biomarker and is also the functional protein of MSCs [25]. These two proteins related with the biological function of MSCs. The expressions of β-catenin and vimentin were evaluated by Western blot analysis. Figure 8 shows that the expression of β-catenin and vimentin of MSCs treated with various concentrations of PBNPs for 48 h had no significant changes compared with the expression of MSCs treated with no PBNPs. These results indicated that PBNPs cannot change the expression of β-catenin and vimentin of MSCs, which showed the stability of biological function of MSCs after treatment with PBNPs.

### In Vitro MRI

MSCs, like other stem cells, have the potential to differentiate bone, cartilage, fat, muscle, and cardiac cells, which have become an important source for regenerative medicine. And tracking the migration and outcome of MSCs is so important to evaluate the role and outcome of exogenous MSCs in defect repair. Magnetic resonance imaging (MRI) helped to observe MSCs after clinical administration, and MRI contrast agent is necessary. The potential of PBNPs used as an effective T2-weighted cellular MRI contrast agent has been demonstrated [14], and some other studies also demonstrated the surface modification of PBNPs enhances its performance in MRI [17, 26]. Currently, PBNP labeling has been used in a variety of cells. Dumont et al. described PBNPs as agents for MRI and fluorescence-based imaging of pediatric brain tumors [27]; Perera et al. developed the gadolinium-incorporated PBNPs for the early detection of tumors in the gastrointestinal tract [28]; and Cano-Mejia et al. combined Prussian blue nanoparticle (PBNP)-based photothermal therapy (PTT) with anti-CTLA-4 checkpoint inhibition to treat neuroblastoma [29]. However, there is rarely related reports on PBNP-labeled MSCs. Additionally, whether there is any negative influence on cell function and viability of MSCs after labeling PBNPs remains unclear

**Fig. 9:**
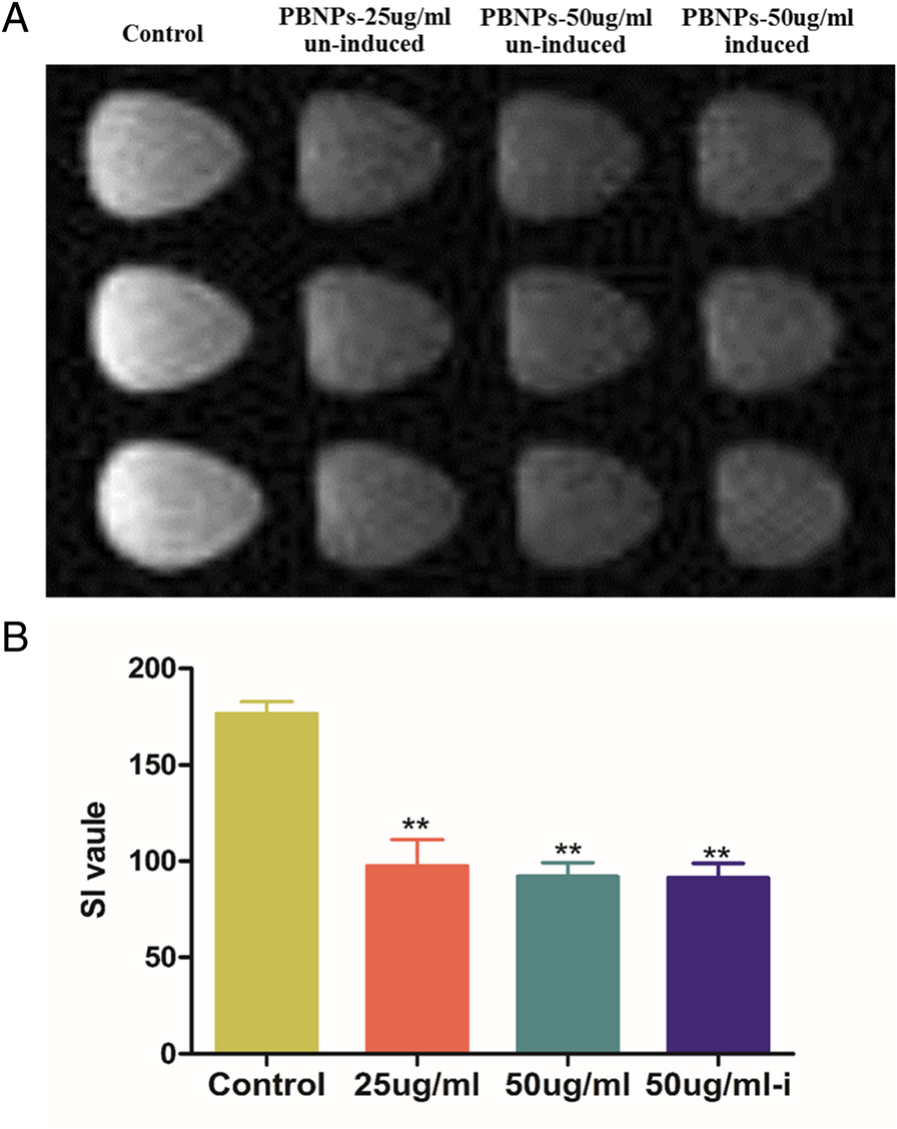
T2-weighted MRI phantoms of MSCs. **a** Transverse section. **b** Quantitative analysis of SI value. **P < 0.001 vs control

To investigate whether the PBNPs have the ability to enhance the T2-weighted MRI contrast of cells, we incubated the MSCs with or without PBNPs and examined the MRI signal effect. To monitor the temporal stability of labeling and to investigate whether the PBNPs would lose its imaging capability when the MSCs differentiated, we incubated the MSCs with PBNPs and induced the MSCs to osteogenic differentiation for 14 days then examined the MRI signal effect. As shown in Fig. 9, the pellets of the MSCs incubated with PBNPs showed a clear MRI signal darkening effect and the SI value of the labeled MSCs had obvious difference with unlabeled MSCs. Notably, the labeled MSCs also showed a clear MRI signal darkening effect when differentiation is induced. These results demonstrated that PBNPs had the potential to be used as an effective T2 contrast agent for cellular imaging of MSCs and can offer long-term retention of the contrast agent even after the cell differentiation.

There are many published data of MSCs labeled with magnetic nanoparticles (MNPs), but the application of MNPs was limited by their cytotoxicity. When delivering MNPs to target tissue, the majority of MNPs often distribute in the liver and spleen, so the toxicity of MNPs cannot be neglected [30]. For example, Costa C found that SPIONs could produce cytotoxicity to neuronal cells and glia cells [31]. As we mentioned above, the PBNPs showed no detectable cytotoxicity and had no effects on the cell characteristics of MSCs including cytoskeleton, cellular morphology, and functional protein. Thus, the strength of using PBNPs as an effective T2-weighted cellular MRI contrast agent would be demonstrated in terms of the cytotoxicity.

## Conclusions

In summary, we introduced the PBNPs to the tracking of mesenchymal stem cells and studied the survival, migration potential, and cell characteristics of MSCs after being labeled with the PBNPs. Furthermore, we also demonstrated the potential of PBNPs as an effective T2-weighted MRI contrast agent for the cellular MRI of MSCs. PBNPs can be effectively used for the labeling of MSCs and will not influence the biological characteristics of MSCs. This conclusion paved a new road for the label of MSCs.

## Abbreviations

DMEM: Dulbecco’s modified Eagle’s medium
DMSO: Dimethyl sulfoxide
FBS: Fetal bovine serum
MRI: Magnetic resonance imaging
MSC: Mesenchymal stem cell
NIR: Near infrared
OD: Optical density
PBNP: Prussian blue nanoparticle
TEM: Transmission electron microscopy
VSM: Vibrating sample magnetometer
